# Introducing an E-learning Solution for Medical Education in Liberia

**DOI:** 10.29024/aogh.21

**Published:** 2018-04-30

**Authors:** S. Walsh, M.R. de Villiers, V.K. Golakai

**Affiliations:** 1 Faculty of Medicine and Health Sciences, Stellenbosch University, ZA; 2 College of Health and Life Sciences, University of Liberia, LR

## Abstract

**Background:**

The Ebola virus epidemic and civil war in Liberia left the country in need of strengthening the health workforce. E-learning in medical education provides relevant learning opportunities for students, develops faculty competencies, and assists with the retention of healthcare workers. The Stellenbosch University Rural Medical Education Partnership Initiative (SURMEPI), the College of Health and Life Sciences (COHLS) at the University of Liberia (UL), and the Health Resources and Services Administration (HRSA) formed a partnership to create an e-learning solution for the COHLS.

**Objective:**

This article outlines the implementation of an e-learning solution for the COHLS in Monrovia, and describes the challenges met, the key successes achieved, and the lessons learnt.

**Methods:**

An initial scoping visit to Liberia was followed by three further on-site visits. Problems identified were: very limited or no network and computer resources, no internet connection, intermittent power, and lack of IT skills. We followed an evolutionary approach to infrastructure implementation by trying various solutions before settling on an offsite-hosted solution using Software as a Service (SaaS). Local staff were upskilled to administer this while remote support from Stellenbosch IT was provided. A stable internet connection was established. Staff and students can access the Learning Management System (LMS) 24/7 using mobile devices and laptops. Workshops were held where staff were taught how to produce online teaching material. Each class has at least one teaching assistant to assist lecturers with uploading and indexing material on the LMS.

A benchmarking visit by COHLS faculty to Stellenbosch University took place, during which an e-learning strategic plan was drawn up. Further online workshops were conducted, and teaching materials were placed on the new LMS.

**Outcomes:**

The intranet that was established consisted of internet connection and software as a service in the form of Office 365, providing access to several products and services. The e-learning model attended to technology and human resources simultaneously. The e-learning strategy aimed to improve teaching and learning at the COHLS, boost the number of qualified doctors, reduce the workload on lecturers, and be scalable in the future.

**Conclusion:**

It is challenging to implement e-learning in medical education. Inadequate infrastructure, limited bandwidth, lack of skilled IT staff, unreliable power supply, time commitment, and ongoing maintenance all need to be overcome. The creation of an e-learning solution for the COHLS over a period of 15 months was enabled by the common vision and close collaboration of the three partners. This model can potentially be replicated across other faculties in the University of Liberia and other educational institutions in Liberia.

## Background

Liberia’s health workforce has faced challenges since the late 1970s when the country’s economic growth slowed, and people emigrated in search of better opportunities. The civil war from 1989–2003 left the health system devastated, destroying infrastructure and causing healthcare worker shortages. In 2006, there were 20 physicians left in Liberia, as opposed to 237 before the war. Only a few training institutions remained, which had limited resources and few academics [1]. Several reforms were instituted under the Sirleaf administration after 2006 to address health workforce shortages, including the reintroduction of free medical education. These strategies resulted in an increase in the numbers of healthcare workers trained, but there remained a maldistribution of healthcare workers between rural and urban areas [1].

During the rebuilding of the health workforce, the Ebola virus epidemic struck in 2013. Health workers were disproportionally affected by the epidemic with deaths reported in two-thirds of those health workers that became infected [2]. The country’s Investment Plan for Building a Resilient Health System in Liberia 2015–2021 highlighted the government’s priorities in rebuilding the health system to ensure it has the capacity, not only to provide the expected essential health services for the people of Liberia, but also to identify and appropriately respond to future health threats [3].

In 2016, the number of registered medical practitioners in active practice in Liberia was 165. This translated into a doctor/patient ratio of 1:25,000 (population 4,000,000) as compared to the World Health Organization recommended ratio of 1:15,000.

There is one medical school in Liberia, namely the College of Health and Life Sciences (COHLS), previously the AM Dogliotti School of Medicine (AMDCM), at the University of Liberia (UL), in Monrovia. The college has a five-year medical pre-service program modelled on the United States of America’s training model. The AMDCM has graduated 392 medical practitioners since its inception in 1968, with continued growth in the past ten years [4]. The current average output is 40 medical graduates per year [3]. The total student enrolment for 2015–2016 was 201 students [4]. There are 31 instructors at the College, of which about 50% are part-time [4].

Curriculum renewal and upscaling of student numbers poses a particular challenge on the African continent, given its inordinate burden of disease and critical shortage of healthcare professionals and medical teachers [5,6]. There is therefore an urgent need to strengthen both the educational facilities and enable the teaching faculty to provide relevant and quality medical education [7,8]. There is literature suggesting that e-learning can address barriers to training and improve retention of health workers [9]. Using e-learning in medical education helps provide relevant learning opportunities for students, as well as developing faculty competencies [9].

SURMEPI formed part of the greater MEPI initiative to improve human resources for health in Africa [6]. SURMEPI developed innovative medical education models in order to strengthen medical education and health systems in rural and resource-constrained environments [10]. One of these models was the development and implementation of e-learning in medical education [11,12]. As a result, the COHLS approached SURMEPI to assist them to create an e-learning solution for them.

The objective of this article is to outline the development and implementation of an e-learning solution for the COHLS. Key successes and lessons learnt are described, which may be useful to other training institutions in resource-constrained environments.

## Methods

An initial scoping visit took place in February 2016 and a Memorandum of Understanding between Stellenbosch University (SU) and the UL was signed. This was followed by three further visits to conduct training workshops in Monrovia and install the IT hardware and software (May 2016, October 2016, and May 2017). A benchmarking visit to the SU Faculty of Medicine and Health Sciences (FMHS) by a delegation from the COHLS took place in November 2016, during which an e-learning strategic plan for the COHLS was drawn up. Online workshops were conducted for lecturers during January 2017. A collection of clinical videos and other teaching material was placed on the new SharePoint server, which has a specially programmed interface that functions as a learning management system (LMS).

### Baseline Visit (February 2016)

The baseline visit followed a series of communications between the COHLS, SU and HRSA. A programme was set for the visit but had to be adapted due to the realities at the host institution. At the time, there was no internet connection at the COHLS, but about 79% of the lecturers and students had a laptop or personal computer (PC). There was no Wi-Fi in the teaching complex or student residences. Many students lived off-campus, also with little internet access. There were around 70 refurbished laptops that were meant to be used by the students. There was one part-time information technology (IT) person on site for the faculty.

Although some basic infrastructure was initially in place (Table 1), most of the whiteboards were unusable due to bad surface cracks, and the data projector was seldom used. Table 1 provides a list of the COHLS existing equipment and facilities in February 2016.

**Table 1:** Existing Equipment and Facilities (Feb 2016).

Facility	Capacity	Existing Equipment

Auditorium (main lecture room)	Seating for 50 to 60 students	Whiteboard. Data projector only on request
Annex Classrooms (3)	Seating for 20 to 30 students	Whiteboards with badly cracked surfaces
Students’ Computer Laboratory	Twenty PCs on an intranet	Running Linux with OpenOffice
Lecturers’ Computer Laboratory	Five PCs on an intranet	Running Linux with OpenOffice
Dell Server	72 terabyte storage	Moodle with some lesson content (not regularly updated)
Wi-Fi Network	Not functioning	
Library	About 6,000 medical and pharmacy books	Manual stock and loan system. Open from 8 am to 4 pm weekdays only

The dean (Prof Golakai) identified three main priorities during the baseline visit:

- Faculty were to be introduced to e-learning and assisted to develop their own electronic lesson material;
- Students needed access to electronic textbooks both while studying and after graduation;
- Internet connectivity to remote hospitals would be useful for telemedicine (so a doctor can get assistance with clinical cases) and to be used for staying up-to-date.

### Proposed Solution to Support E-learning

**Figure 1:**
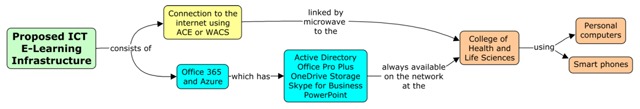
Proposed E-learning Infrastructure.

At the end of the baseline visit, it was clear that the infrastructure required for the COHLS needed the input of a systems architect. A senior systems analyst from SU was then brought on board. At this stage, the plan was (Figure 1):

- To establish an internet connection;
- To install a server which could be remotely configured and controlled;
- To acquire Microsoft Office 365 [13] licenses for students and lecturers;
- To use TechSmith Relay [14] for screen casting lectures (subsequently dropped as the lack of on-site IT expertise became apparent);
- To enable videoconferencing.

The intention was to turn the college into an e-teaching and e-learning environment and upskill all IT staff, lecturers and students. This required access to email, authenticated login, cloud backup, Windows, all Microsoft programs and other software. The expertise to run much of this would be supplied remotely by SU IT, thus addressing the lack of IT skills available locally at the time. It was important to implement the system in small steps to determine what worked and what did not. Implementation was planned as a phased approach consisting of three visits to COHLS, during which time equipment was supplied, installed, configured, and tested. At the same time, capacity development workshops were conducted for lecturers and students. Much configuration of the equipment was done remotely once the COHLS was connected to the internet. Training was provided for the local IT staff.

### Installing the Internet Connection

During the civil wars, most of Liberia’s communications infrastructure was destroyed, so fixed landlines (digital subscriber lines) could not be used. Cell phone coverage in Monrovia was reasonable through three main suppliers (LibTelco, CellCom, and LoneStar). Cellular data was expensive and sometimes not available even in urban areas.

Liberia is connected to the Africa Coast to Europe (ACE) fibre-optic undersea cable that comes aground in Monrovia and is controlled by a public-private group called the Cable Consortium of Liberia. Because of the high bandwidth costs and the unavailability of funds, the UL had no permanent internet connection.

After thoroughly exploring various options including cellular, satellite, and microwave links, last-mile internet was secured through PowerNet, who provided a 10-megabyte fully redundant connection. They were directly linked to ACE and offered continuous support all year round. Communication with distant hospitals could be facilitated in the future as the company had ample experience with satellite connections and could assist with connecting remote locations.

A Wi-Fi network connected to the internet was installed and covered the lecture halls and administrative building. The signal was strong enough for staff and students to be able to have internet access from the buildings across the road from the annex (i.e. the Physiology and Anatomy laboratories).

### Installing the Hardware and Software

Much of the equipment had to be acquired outside of Liberia and couriered to the country. This necessitated engaging with complex procurement and delivery systems in the sending country and import control regulations in Liberia. The equipment had to be classified as donated to comply to import tax regulations.

Four data projectors were permanently installed in the lecture rooms – a major undertaking that took more than a week to accomplish. All the power plugs in the main auditorium and three annex classrooms were replaced, as most were not functioning. Students could then bring their laptops to class and take notes during lectures. Crack-filler for wall defects, primer, brushes and special white paint for coating the projection walls in the classrooms were supplied to renovate facilities. Pilot testing of the videoconferencing facility in the Lecturers Laboratory over the internet connection worked well during the on-site visits. Table 2 lists the hardware and software that was installed at the COHLS over 12 months.

**Table 2:** ICT Infrastructure Installed (May 2016 to May 2017).

Hardware	Software

Dell PowerEdge Server, uninterruptable power supply (UPS) and switch.	Microsoft Office 365 licenses for 450 students and lecturers.
Five data projectors (one for each lecture room and one spare) with five spare bulbs.	Special interface in SharePoint to simulate a LMS.
55-inch monitor screen mounted on the wall of Lecturer’s Laboratory.	SharePoint configured with COHLS course outline structure and populated with existing as well as new e-learning content.
Jabra omnidirectional microphone and Logitech webcam.	CmapTools [15] (Concept map).
Lecture hall loudspeaker system consisting of two remote handheld microphones, internal battery supply, Bluetooth connections and internet connectivity.	

### Capacity Building Workshops

Initially there was poor understanding of e-learning and little familiarity with software, although most had used Microsoft Office (Word and PowerPoint) to some degree. Over the twelve months of the project, eight workshops on various e-learning topics were held during each of the three site visits (workshops were duplicated to allow more to attend). These were run while the IT equipment was being installed. Three additional online workshops were also done. The aim of the workshops (Table 3) was to make lecturers comfortable with using technology for both synchronous and asynchronous teaching. A total of 160 participants attended the workshops (approximately 70% lecturers and the rest teaching assistants/senior students). All lecturers/module chairs were expected to attend and computer-literate students from each class were selected for training. In addition to these workshops, regular training sessions were held for the COHLS IT support person at least once weekly throughout the entire project.

**Table 3:** Workshops.

Workshop Topic	Software Used	Application for the COHLS

Concept Mapping for Knowledge Transfer	CmapTools	Empowers users to construct, navigate, share, and criticize knowledge models represented as concept maps. Attendees got practical experience on how to create concepts maps of their lectures. These can be used by lecturers for teaching and evaluation and by students for learning.
Creating Sreencasts/Videos Using PowerPoint	Office 2013 or 2016 with PowerPoint	Attendees got practical experience on creating their own screencasts/videos of their lectures using PowerPoint. We covered some PowerPoint’s basics and progressed to recording the lecturer’s voice over their slides. Each person was given a headset microphone. We concluded with producing a screencast or video of a PowerPoint lesson.
Finding Evidence-based Medical Information on the Internet	Laptop with a browser and internet connection.	Hands-on experience on how to find evidence-based information on the internet. The workshop covered Boolean logic and progressed to using Medical Subject Headings (MesH) for retrieving results from PubMed and its related medical databases. We concluded with an overview of a wide range of open source online journals.
Using OneNote and Class Notebooks	Microsoft OneNote and Class Notebook	Lecturers and Teaching Assistants were taught to use the tools on Office 365 in for creating online lessons for uploading to their LMS. Online sources of free, peer-reviewed medical courses were also evaluated for possible inclusion in their modules.
Medical Apps for Your Smartphone	Online workshops with supporting PDF and video.	Lecturers attended the online workshops on using various useful apps on their smartphones to support patient care. Several apps were demonstrated, including a drug database and HIV treatment guidelines.

**Figure 2:**
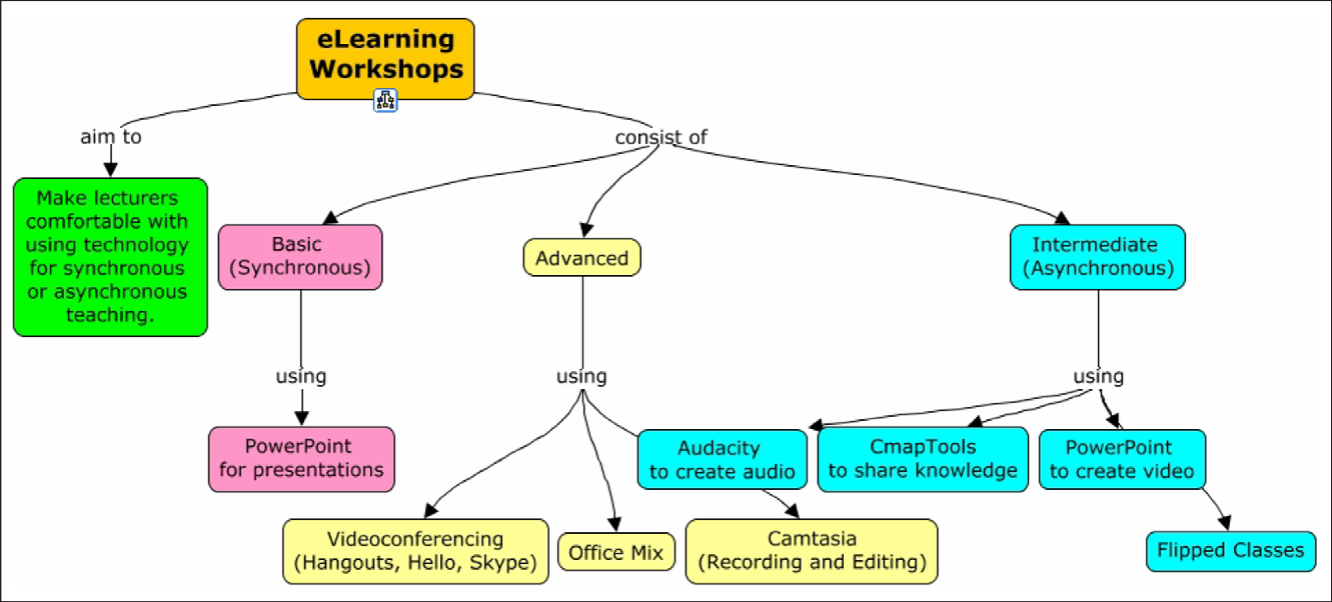
Capacity Building Workshops.

The workshop participants brought their own laptops. Browsers and Office suites were upgraded an additional software such as concept maps (CmapTools) [15] installed. It was evident in the workshops that the level of computer literacy in faculty was initially generally low. This was attributed to the lack of a Wi-Fi network, expensive data and no internet connectivity. Most had outdated operating systems and Office products. Few participants used a mouse, which lead to difficulties in working with complex programmes such as creating Cmaps. It was not easy to schedule workshop times as potential attendees were very busy and weekends were inconvenient. Figure 2 shows the content and approaches that were conveyed during the workshops.

The lecturers wanted further workshops but due to unfamiliarity with the range of possibilities, struggled to suggest their own topics of interest. As medical apps for a smartphone could be extremely useful, a video was created documenting various medical apps, which was uploaded to the SharePoint LMS site as an example of online content [16].

Anonymous online surveys conducted after the workshops was positive. Participants felt the content was well presented and that the practical demonstrations and hands-on approach was helpful to master key IT skills. They would recommend the workshops to colleagues and intend to use what they learnt in their workplace. The participants wanted more time for the workshops and requested regular refresher courses going forward. In terms of the online workshops, they were satisfied with the support received and that the video-conferencing facilities worked well.

### Benchmark Visit (November 2016)

Ten delegates from the COHLS visited the FMHS at SU in South Africa during November 2016. Apart from the lecturers, the delegation included both the interim dean and the recently retired dean, a medical student and a post-graduate student. SU FMHS e-learning facilities were visited and various activities and strategies for e-learning in medical education were discussed with the delegation. A full day was devoted to developing a strategic plan for the implementation of e-learning at the COHLS.

## Outcomes

### Office 365 Functionality for the COHLS

**Figure 3:**
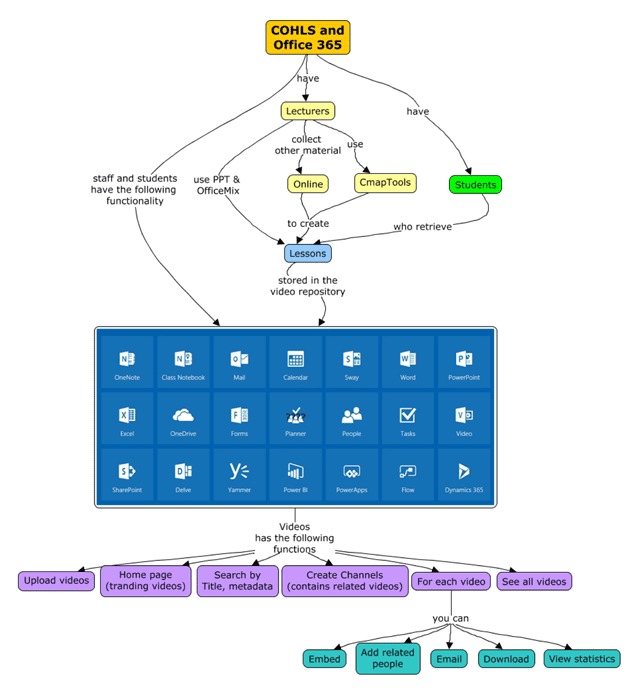
Overview of Office 365 Functionality for the COHLS.

The intranet that was established at COHLS had an internet connection and “software as a service” (SaaS) in the form of Office 365, providing access to several products and services with a single sign-on. This approach was important in a resource-constrained environment as it reduced the need for specialised on site IT skills, whilst still providing a first-world e-learning environment that is available to students and staff anywhere and at any time. Figure 3 provides an overview of the Office 365 functionality that was now available to students and staff 24/7.

### E-learning Model

**Figure 4:**
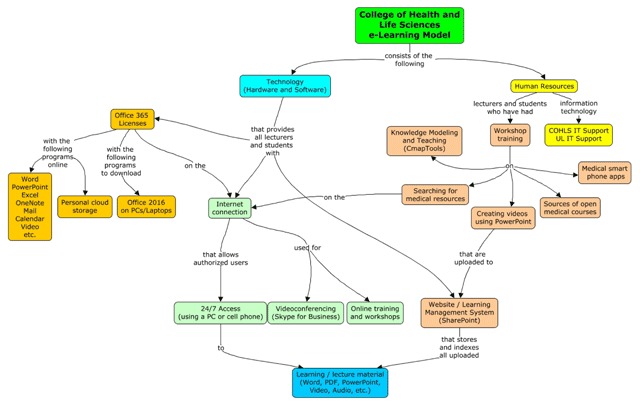
The E-learning Model.

The e-learning model that was created for the COHLS targeted technology and human resources simultaneously. Staff and students assimilated a variety of topics that enabled them to use the new technology. Material from the workshops was uploaded onto the new LMS to be available for updating knowledge and skills. A local IT support person was equipped to maintain the new system. All students and staff at the COHLS now have free access to the internet at the medical campus. This allows them to access and download the teaching material on the LMS SharePoint server (or any internet resource) any time of the day or night. They also have access to Office 365 on their PCs, laptops, or smartphones and can download the full Office 2016 suite. Learning material can be stored in the cloud (on OneDrive), or downloaded locally and taken off campus. See Figure 4.

### The Strategic Plan

The strategic plan for e-learning at the COHLS envisaged that the implementation of the e-learning strategy would improve teaching and learning at the COHLS with outreach to other parts of Liberia, create IT competent COHLS end-users, and propel the COHLS and UL into the digitalized 21st century. Strategies that were set out to achieve these outcomes were the following:Perform curriculum content and delivery review to enable the use of e-learning.

- Identify and use instructors and student peers to teach IT skills and content creation.
- Make technology affordable and accessible to students and lecturers.
- Use online resources.
- Motivate students and lecturers to use e-learning facilities.
- Take all new students through basic IT literacy training.
- Generate an online repository of learning and assessment materials.

## Discussion

The creation of an e-learning solution for the COHLS over a period of 15 months was enabled by the common vision and close collaboration of the three partners (COHLS, SU and HRSA). There was enthusiastic buy-in from the UL and the COHLS. All the work done was based on COHLS priorities and adapted to suit the local context. The COHLS has made a quantum leap forward in terms of their IT equipment, internet connectivity, and students’ and staff’s IT capacity. The COHLS now have a functioning e-learning system and a strategic plan to take them forward. Staff and students have continuous access to the internet and LMS. Videoconferencing, email addresses, and personal cloud storage accessible by PC or smartphones are all freely available. This model can potentially be replicated across UL and other teaching institutions in the country.

It is challenging to implement e-learning in medical education, not only in lower middle-income countries (LMICs) [9]. Inadequate infrastructure, limited bandwidth, lack of skilled IT staff, time commitment, and maintenance of e-learning are all factors that demand attention [9]. Daily power outages in Monrovia disrupted communication, impeded on the availability of internet for staff and students and was potentially damaging to electronic equipment. Close collaboration was needed to overcome communication challenges.

Sustainability is a major consideration as the high cost of internet access is a potential problem. It is a priority for educational institutions in Liberia to establish a National Research and Education Network (NREN), which is usually a non-profit organization functioning with government backing but working independently to negotiate bandwidth prices with internet service providers. Two fibre-optic undersea cables could be used, namely the ACE or the WACS (West Africa Cable System). A group to the east of Liberia called WACREN [17] (West and Central African Research and Education Network) uses the WACS internet connection. Recently Sierra Leone took its first steps toward establishing an NREN [18]. NRENs have proven successful in driving down the cost of internet for educational and research purposes, possibly by 50% or more. Recently Google has been appointed to create a fibre-optic ring around Monrovia but commercial concerns prevent the COHLS from benefiting.

The Office 365 licence fees need to be paid annually. Fortunately, being an educational institution means that the UL can obtain this at a greatly reduced fee. It would be good to get other departments at UL to use the same platform as much as possible to reduce cost due to duplication. It is best to use hosted services (such as SaaS) where possible given the lack of local expertise.

Touray et al. identified 43 barriers in implementing ICT in LMIC countries [19]. These were grouped into eight critical success factors, namely socio-cultural, infrastructural, political and leadership, legal and regulatory, economical, educational and skills, security and safety, and technical [19]. Infrastructural and economic constraints posed the biggest obstacle in their case study, but all these factors need attention. They found country-specific differences in the occurrence of the other factors, which emphasizes the importance of adapting to the context when implementing e-learning.

The success of e-learning greatly depends on good infrastructure that delivers services and makes them readily accessible, which requires a large investment as well as continuous maintenance [9,19]. A larger and more highly trained workforce is needed who have hardware, software, and e-learning training skills. Several valuable lessons were learnt during the planning and implementation of this project (Table 4). These lessons can be of use for setting up e-learning platforms in similar contexts.

**Table 4:** Lessons Learnt.

Close collaboration is needed more so when there are communication challenges. Mainstream funding for maintenance and repairs should be made available. Expensive bandwidth needs to be addressed by a National Research and Education Network (NREN). A knowledgeable and reliable internet service provider is essential. A reliable power supply is a priority as the lack of a stable power supply could severely compromise the impact of e-learning interventions. Capacitating a local on-site person to support and maintain the e-learning model is crucial. The level of computer literacy needs to be assessed. Capacity building training should be adapted to address various levels of competencies. Time for workshops should be reserved as people have busy schedules. Getting to know the issues that really matter to local staff and students is essential as they can be unfamiliar with various e-tools and e-learning.

## Conclusion

The e-learning model at the COHLS has been successfully introduced, but the endeavour is not yet self-sustaining. It was vital to get the infrastructure installed correctly and configured so that the model can be expanded seamlessly. The exciting possibility exists to expand the model to the rest of UL with its approximately 46,000 registered students. Establishing, maintaining, and expanding an e-learning environment at the entire COHLS is a complex task that requires a dedicated team who possess an array of technical skills. The team should include carefully selected consultants whose special expertise can be called on when required. The long-term benefits for the Liberian healthcare system are potentially enormous, and the model used at the COHLS could be emulated at other educational institutions in Liberia.
